# The Roles of Cytochrome *b*_559_ in Assembly and Photoprotection of Photosystem II Revealed by Site-Directed Mutagenesis Studies

**DOI:** 10.3389/fpls.2015.01261

**Published:** 2016-01-12

**Authors:** Hsiu-An Chu, Yi-Fang Chiu

**Affiliations:** Institute of Plant and Microbial Biology – Academia SinicaTaipei, Taiwan

**Keywords:** photosynthesis, photosystem II, cytochrome *b*_559_, site-directed mutagenesis, photoprotection, photoinhibition

## Abstract

Cytochrome *b*_559_ (Cyt *b*_559_) is one of the essential components of the Photosystem II reaction center (PSII). Despite recent accomplishments in understanding the structure and function of PSII, the exact physiological function of Cyt *b*_559_ remains unclear. Cyt *b*_559_ is not involved in the primary electron transfer pathway in PSII but may participate in secondary electron transfer pathways that protect PSII against photoinhibition. Site-directed mutagenesis studies combined with spectroscopic and functional analysis have been used to characterize Cyt *b*_559_ mutant strains and their mutant PSII complex in higher plants, green algae, and cyanobacteria. These integrated studies have provided important *in vivo* evidence for possible physiological roles of Cyt *b*_559_ in the assembly and stability of PSII, protecting PSII against photoinhibition, and modulating photosynthetic light harvesting. This mini-review presents an overview of recent important progress in site-directed mutagenesis studies of Cyt *b*_559_ and implications for revealing the physiological functions of Cyt *b*_559_ in PSII.

## Introduction

**FIGURE 1:**
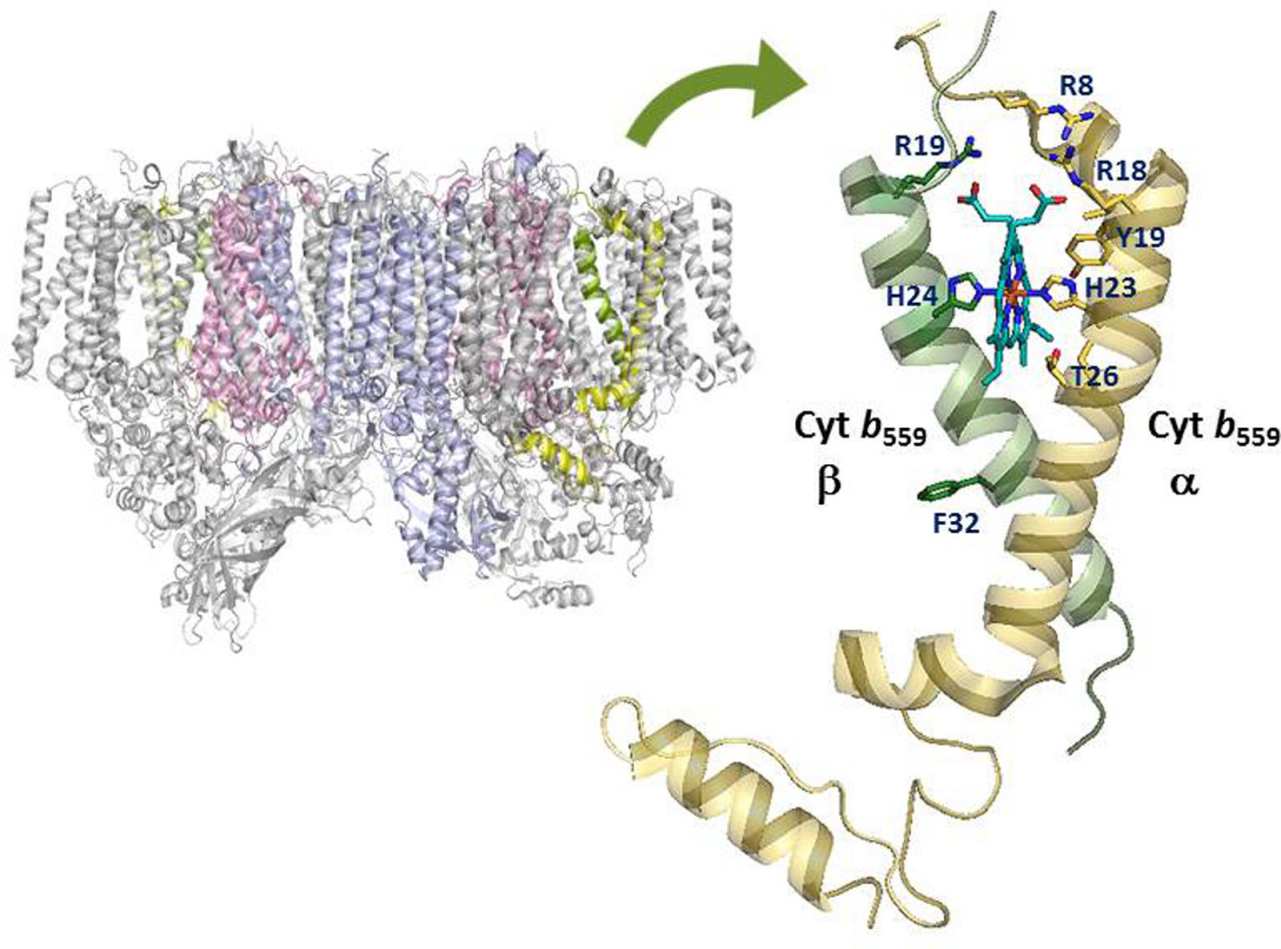
**Structural model of the cytochrome *b*_559_ in Photosystem II**. Amino acid residues of cytochrome *b*_559_ targeted by recent site-directed mutagenesis work are labeled by using amino acid sequences of cytochrome *b*_559_ from *Thermosynechococcus elongatus*. The figure was created using PyMol and the PDB file 4UB8.

Cytochrome *b*_559_ is one of the essential components of Photosystem II in all oxygenic photosynthetic organisms (Whitmarsh and Pakrasi, 1996; Stewart and Brudvig, 1998; Guskov et al., 2009; Umena et al., 2011). Cyt *b*_559_ is a heme-bridged heterodimer protein comprising one α- and one β- subunit (encoded by the *psbE* and *psbF* genes) of 9 and 4 kDa, respectively (see **Figure 1**). Each subunit provides a His ligand (His-22 residue of the α- or β-subunit of Cyt *b*_559_ in *Synechocystis* sp. PCC 6803, corresponding to His-23 residue of α- or His-24 residue of the β-subunit of Cyt *b*_559_ in *Thermosynechococcus elongatus*) for the non-covalently bound heme, which is located near the stromal side of PSII. In addition, Cyt *b*_559_ has different redox potential forms depending on the type of PSII preparations and treatments: a HP form with a midpoint redox potential of about +400 mV, an IP form of about +200 mV, and a LP form with a midpoint redox potential of about 0–80 mV (Stewart and Brudvig, 1998; Roncel et al., 2001 and references therein). In intact PSII preparations, Cyt *b*_559_ is mostly in the reduced HP form under ambient conditions. In inactive or less intact PSII preparations, Cyt *b*_559_ is typically in the LP or IP form and mostly oxidized (presumably by molecular oxygen) under ambient conditions (Barber and De Las Rivas, 1993; Poulson et al., 1995; Pospíšil et al., 2006).

**FIGURE 2:**
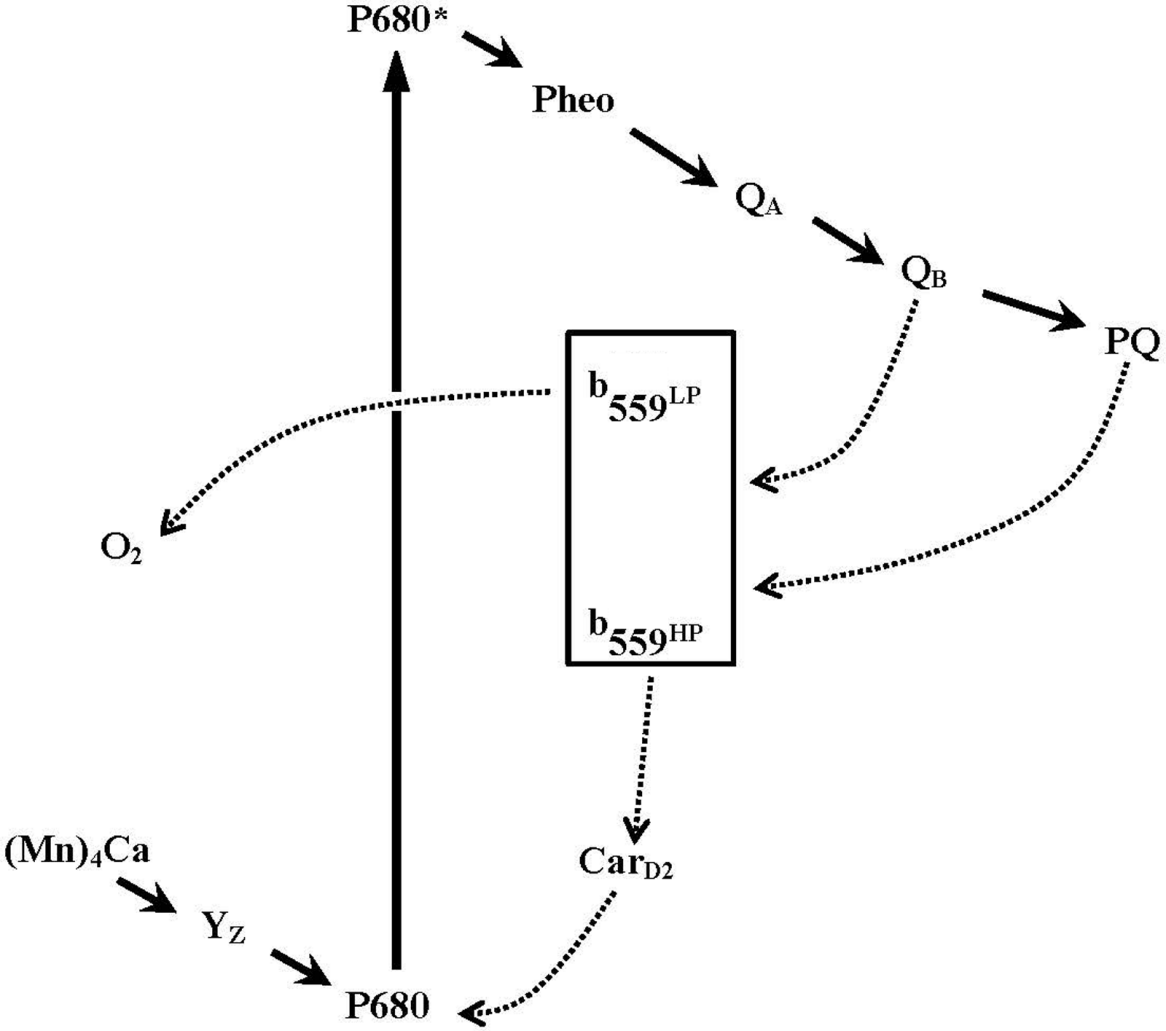
**A simplified scheme for electron transfer pathways within Photosystem II reaction centers**. Primary electron transfer pathway (bold solid lines with arrows) and possible secondary electron transfer pathways involving cytochrome *b*_559_ (dotted line with arrows) are shown. The scheme is modified from Whitmarsh and Pakrasi (1996).

Several studies have proposed that Cyt *b*_559_ participates in secondary electron transfer pathways that protect PSII against photoinhibition (see **Figure 2**; Heber et al., 1979; Falkowski et al., 1986; Thompson and Brudvig, 1988; Barber and De Las Rivas, 1993; Poulson et al., 1995; Magnuson et al., 1999; Faller et al., 2001; Tracewell and Brudvig, 2008 and references therein). In these models, the HP form of Cyt *b*_559_ is thought to donate its electron, via a β-carotene molecule (Car_D2_), to reduce highly oxidized chlorophyll radicals in PSII under donor-side photoinhibitory conditions (e.g., the oxygen-evolving complex is impaired or under assembly). Oxidized Cyt *b*_559_ may accept an electron from the acceptor side of PSII (Q_B_^−^, or reduced PQH_2_ from the pool), thus forming a cyclic pathway of electron transfer within PSII. On the other hand, when the electron transfer on the acceptor side of PSII is inhibited (e.g., under high-light conditions), the oxidized Cyt *b*_559_ might accept an electron from the acceptor side of PSII to prevent the formation of damaging singlet oxygen species (Nedbal et al., 1992; Vass et al., 1992; Barber and De Las Rivas, 1993; Bondarava et al., 2010). In addition, several different enzymatic functions of Cyt *b*_559_ have been proposed, such as superoxide dismutase (Ananyev et al., 1994) and PQH_2_ oxidase in intact PSII (Kruk and Strzałka, 1999, Kruk and Strzalka, 2001; Bondarava et al., 2003, 2010) and superoxide oxidase and reductase in tris-washed PSII (Tiwari and Pospísil, 2009; Pospíšil, 2011). Moreover, a novel quinone-binding site (Q_C_) was identified close (about 15 Å) to the heme of Cyt *b*_559_ in the 2.9-Å PSII crystal structure from *T. elongatus* (Guskov et al., 2009). The occupancy of this Q_C_ site with PQ (or PQH_2_) has been proposed to modulate the redox equilibration between Cyt *b*_559_ and the PQ pool (Kaminskaya et al., 2006, 2007; Kaminskaya and Shuvalov, 2013) or be involved in the exchange of PQ on the Q_B_ site from the pool (Guskov et al., 2009). However, the Q_C_ site was not detected in the more recent 1.9-Å PSII crystal structure (Umena et al., 2011). Despite the recent remarkable progress in understanding the structure and function of PSII, the exact function of Cyt *b*_559_ in PSII remains unclear.

This mini-review gives an overview of important progress in recent site-directed mutagenesis studies performed to reveal the physiological function(s) of Cyt *b*_559_ in PSII. More comprehensive reviews on the structure and functions of Cyt *b*_559_ are available (Whitmarsh and Pakrasi, 1996; Stewart and Brudvig, 1998; Faller et al., 2005; Pospíšil, 2011; Shinopoulos and Brudvig, 2012).

## Function in Assembly and Stability of PSII Reaction Centers

Prior mutagenesis studies with *Synechocystis* sp. PCC 6803, *Chlamydomonas reinhardtii* or *Nicotiana tabacum* showed no stable PSII reaction centers assembled in the absence of either Cyt *b*_559_ subunit (Pakrasi et al., 1988, 1989, 1990; Morais et al., 1998; Swiatek et al., 2003; Suorsa et al., 2004). Several authors proposed that Cyt *b*_559_ plays an important structural role, such as being a nucleating factor, during the early stage of PSII assembly (Pakrasi et al., 1988; Morais et al., 1998; Komenda et al., 2004).

In addition, mutagenesis studies with *Synechocystis* sp. PCC 6803 showed that substituting either of the heme axial ligands (His22 of the α-subunit or His22 of the β-subunit of Cyt *b*_559_ in *Synechocystis* sp. PCC 6803) with Leu, Met, Glu, Gln, Tyr, Lys, Arg, or Cys abolished the photoautotrophic growth and severely diminished the assembly or stability of PSII in the mutant cells, except for H22Kα mutant cells, which were able to grow photoautotrophically and accumulated stable PSII reaction centers (∼81% as compared with wild-type cells; Pakrasi et al., 1991; Hung et al., 2007, 2010). Electron paramagnetic resonance results indicated the displacement of one of the two axial ligands to the heme of Cyt *b*_559_ in H22Kα mutant reaction centers, at least in isolated reaction centers (Hung et al., 2010). In addition, H22Kα and Y18Sα (corresponding to Y19Sα in *T. elongatus*) in mutant PSII core complexes contained predominately the LP form of Cyt *b*_559_. The findings support the concept that the redox properties of Cyt *b*_559_ are strongly influenced by the hydrophobicity and ligation environment of the heme (Krishtalik et al., 1993; Gadjieva et al., 1999; Roncel et al., 2001; Pospíšil and Tiwari, 2010).

Spectroscopic and functional characterizations of the cyanobacterium *Synechocystis* sp. PCC 6803 with mutation of charged residues on the cytoplasmic side of Cyt *b*_559_ in PSII have been reported (Chiu et al., 2013). All mutant cells grew photoautotrophically and assembled stable PSII. However, R7Eα, R17Eα, and R17Lβ mutant cells grew significantly slower and were more susceptible to photoinhibition as compared with wild-type cells. In addition, the PSII core complexes from R7Eα and R17Lβ cells contained predominantly the LP form of Cyt *b*_559_. Electron paramagnetic resonance results indicated the displacement of one of the two axial ligands to the heme of Cyt *b*_559_ in the reaction centers of the R7Eα and R17Lβ mutants. In recent PSII crystal structural models (Guskov et al., 2009; Umena et al., 2011), the side chains of these Arg residues of Cyt *b*_559_ (corresponding to Arg8 and Arg18 residues of the α-subunit and Arg19 residue of the β-subunit of Cyt *b*_559_ in *T. elongatus*) are in close contact with the heme propionates of Cyt *b*_559_ (see **Figure 1**). Thus, the electrostatic interactions between these Arg residues and the heme propionates of Cyt *b*_559_ may affect the ligation structure and redox properties of the heme in Cyt *b*_559_ (Chiu et al., 2013).

Furthermore, mutagenesis studies of *C. reinhardtii* showed that the H23Yα, H23Mα, and H23Cα mutant cells were unable to grow photoautotrophically, were sensitive to photoinhibition, accumulated 10–20% of the PSII (compared to wild-type cells), and contained a disrupted heme pocket while still retaining significant O_2_ evolution activity (Morais et al., 2001; Hamilton et al., 2014). Thus, the heme of Cyt *b*_559_ was not required for photosynthetic water oxidation by PSII (Morais et al., 2001). A recent study also presented evidence to ascribe the photoinhibition phenotype of H23Cα mutant cells to a faster rate of photodamage and an impaired PSII repair cycle (Hamilton et al., 2014). Hence, Cyt *b*_559_ may play important roles in the assembly, repair and maintenance of the PSII complex *in vivo*.

In the other recent mutant study of *T. elongatus* that took advantage of the robustness of the PSII variant with PsbA3 as the D1 subunit, the four constructed Cyt *b*_559_ mutants (H23Aα, H23Mα, Y19Fα, and T26Pα) grew photoautotrophically (*T. elongatus* is an obligate photoautotroph; Sugiura et al., 2015). Although the H23Aα and H23Mα mutants assembled only an apo-Cyt *b*_559_, the steady-state level of active PSII was comparable to that in the wild-type control. The results suggest that the heme has no structural role in the assembly of PSII in the presence of α- and β-subunits of Cyt *b*_559_. This finding is in strong contrast to the *Synechocystis* sp. PCC 6803 mutant showing that proper coordination of the heme cofactor in Cyt *b*_559_ is important to the assembly or stability of PSII (Pakrasi et al., 1991; Hung et al., 2007). In addition, Cyt *b*_559_ mutant cells of *T. elongatus* showed no correlation between the rate of photoinhibition and the redox potential of the heme. However, the recovery of the oxygen-evolving activity of PSII after photoinhibition was significantly slower in these mutant cells. PsbA3 is the D1 isoform expressed in *T. elongatus* under high-light conditions (Nakamura et al., 2002; Kós et al., 2008). The high-light D1 isoform in cyanobacteria has a Glu instead of a Gln residue (for the low-light D1 isoform) at position 130 in the D1 protein sequence (for a review, see Mulo et al., 2009) and this Glu residue forms hydrogen-bonding interactions with pheophytinD1 (Dorlet et al., 2001; Shibuya et al., 2010). Cyanobacterial PSIIs with the high-light D1 isoform showed increased photo-tolerance and accelerated non-radiative charge recombination (Tichy et al., 2003). This phototolerant property has been attributed to a photoprotection mechanism involving the redox potential of pheophytinD1, which enhances the probability for non-radiative recombination of the singlet radical pair and prevents the formation of potentially damaging ^3^P680 and singlet oxygen species (Vass and Cser, 2009; Sugiura et al., 2014). Further investigation could determine whether PsbA3 may compensate the photoprotective function of Cyt *b*_559_ in the assembly and stability of PSII in these Cyt *b*_559_ mutant cells.

## Function in Protecting PSII Against Photoinhibition

Numerous site-directed mutagenesis studies have investigated the role of Cyt *b*_559_ in protecting PSII against photoinhibition under high light. In the photoprotective models, oxidized Cyt *b*_559_ may accept an electron from the acceptor side of PSII (Q_B_^−^, Q_C_, or reduced PQH_2_ from the pool). Previous mutant studies of dark-adapted leaves of the F26Sβ Cyt *b*_559_ tobacco mutant showed a greatly reduced PQ pool, conversion of the redox-potential form of Cyt *b*_559_ to the LP form, and photosynthetic activities sensitive to high light (Bondarava et al., 2003, 2010). In addition, R7Eα and R17Lβ Cyt *b*_559_ mutant cells of *Synechocystis* sp. PCC 6803 and R18Sα Cyt *b*_559_ mutant cells of *T*. *elongatus* showed markedly reduced PQ pools, altered redox-potential forms of Cyt *b*_559_, and high susceptibility to light stress (Chiu et al., 2013; Guerrero et al., 2014). A defect in PQH_2_ oxidase activity of Cyt *b*_559_ due to altered redox-potential forms of Cyt *b*_559_ in these mutant strains could explain their high susceptibility to strong light and greatly reduced PQ pools. Therefore, Cyt *b*_559_ may function as a PQH_2_ oxidase to keep the PQ pool and the acceptor side of PSII oxidized in the dark, thereby preventing PSII from acceptor-side photoinhibition (Kruk and Strzałka, 1999, Kruk and Strzalka, 2001; Bondarava et al., 2003, 2010). However, one recent study reported no defect in PQH_2_ oxidation in the dark in H23Cα mutant cells of *C. reinhardtii*, even though H23Cα mutant cells contained a disrupted heme-binding pocket of Cyt *b*_559_ and were sensitive to photoinhibition (Hamilton et al., 2014). Further studies are required to clarify this discrepancy. In addition, study of the 2.9-Å resolution PSII crystal structure reported the binding of Q_C_ at a hydrophobic cavity near Cyt *b*_559_ (Guskov et al., 2009). Several spectroscopic studies have provided evidence that the occupancy of the Q_C_ site by PQ (or PQH_2_) may modulate the redox potential of Cyt *b*_559_ and mediate the redox equilibration between Cyt *b*_559_ and the PQ pool (Kaminskaya et al., 2006, 2007; Kaminskaya and Shuvalov, 2013). A recent study provided evidence of a possible one-electron oxidation of PQH_2_ by Cyt *b*_559_ at the Q_C_ site involved in the formation of a superoxide anion radical (Yadav et al., 2014). The above results are consistent with Cyt *b*_559_ possibly accepting an electron from PQH_2_ via the Q_C_ site in PSII. However, the Q_C_ site was not present in the more recent 1.9-Å PSII crystal structure (Umena et al., 2011). Further investigations are needed to solve this important issue.

Spectroscopic and functional characterization of the H22Kα and Y18Sα Cyt *b*_559_ mutant cells of *Synechocystis* sp. PCC 6803 showed that both mutants have functional PSII and exhibited the normal period-four oscillation in oxygen yield (Hung et al., 2010). However, both mutants were more susceptible to photoinhibition than the wild type under high-light conditions. In addition, PSII core complexes from the H22Kα and Y18Sα mutants predominantly contained the oxidized LP form of Cyt *b*_559_ (∼79 and 86%, respectively). A defect in the photoprotective function of Cyt *b*_559_ in H22Kα and Y18Sα mutants could explain their high susceptibility to strong light. Furthermore, H22Kα and Y18Sα Cyt *b*_559_ mutants in a D1-D170A genetic background that prevented assembly of the Mn cluster showed almost completely abolished accumulation of PSII even under normal-growth-light conditions. The data support an important redox role of Cyt *b*_559_ in protecting PSII under donor-side photoinhibition conditions (Hung et al., 2010).

Furthermore, under low light, the H23Cα Cyt *b*_559_ mutant showed more rapid assembly of the Mn_4_CaO_5_ cluster than the wild-type control in *C. reinhardtii* (Hamilton et al., 2014). However, the photoactivation of oxygen-evolving PSII in the H23Cα mutant was inhibited under high light. The results suggest that reduction of P680^+^ via cyclic electron flow within PSII (via Cyt *b*_559_ and Car_D2_, **Figure 2**) may compete with the photoactivation process and provides important *in vivo* evidence for a photoprotective role of Cyt *b*_559_ in photo-assembly of the Mn_4_CaO_5_ cluster in PSII (Hamilton et al., 2014).

A recent mutant study involving *T. elongatus* showed that the midpoint redox potential of the HP form of Cyt *b*_559_ was significantly destabilized (converted to the IP form) in mutant PSII core complexes of Cyt *b*_559_ mutant strains (I14Aα, I14Sα, R18Sα, I27Aα, I27Tα, and F32Yβ; Guerrero et al., 2014). When the oxygen-evolving complex was inactive, the yield of dark-reduction of Cyt *b*_559_ was lower and the kinetics was slower in the R18Sα mutant than in wild-type cells. The results support the concept that the HP form of Cyt *b*_559_ may function as a PQH_2_ oxidase to keep the PQ pool oxidized and also as an electron reservoir for the cyclic electron flow within PSII when the donor-side of PSII is impaired (Guerrero et al., 2014).

Moreover, a previous spectroscopic study showed that different spectral forms of Car were oxidized in PSII samples containing different redox forms of Cyt *b*_559_ (Tracewell and Brudvig, 2008). The authors proposed that the quenching properties of PSII may be controlled by the redox form of Cyt *b*_559_ by modulating the different type of oxidized Car species (radical cation or neutral radical) formed in PSII. Future study could investigate the quenching properties of PSII in the wild type versus Cyt *b*_559_ mutant strains of cyanobacteria with different redox forms of Cyt *b*_559_ to validate this proposal.

## Effects on Photosynthetic Light Harvesting

Recent mutant studies revealed a novel role of Cyt *b*_559_ in modulating photosynthetic light harvesting in PSII reaction centers. A spontaneously generated mutant from *Synechocystis* sp. PCC 6803 wild-type cells grown in BG-11 agar plates containing 5 mM Glu and 10 μM DCMU carried an Arg7 to Leu mutation on the alpha-subunit of Cyt *b*_559_ in PSII (Chiu et al., 2009). Results of 77-K fluorescence and room-temperature chlorophyll *a* fluorescence spectra indicated that the energy transfer from phycobilisomes to PSII reaction centers was partially inhibited or uncoupled in this mutant. In addition, the cytoplasmic side of Cyt *b*_559_ is located within the predicted contact sites in PSII for the APC core complex of the phycobilisome (Barber et al., 2003). The Arg7 to Leu mutation of Cyt *b*_559_ may alter the interaction between the APC core complex and PSII reaction centers, thereby reducing energy delivery from the antenna to the reaction center and protecting mutant cells against DCMU-induced photo-oxidative stress (Rutherford and Krieger-Liszkay, 2001).

Many cyanobacteria including *Synechocystis* sp. PCC 6803 have a novel blue-green light-induced NPQ mechanism to protect PSII reaction centers against photodamage under high-light stress (Kirilovsky and Kerfeld, 2012). Under high-light conditions, a soluble orange carotenoid protein is able to absorb blue–green light and undergoes photo-conversion into the active red form, which interacts with the APC core of the phycobilisome and dissipates excess excitation energy from the phycobilisome as heat. Interestingly, several *Synechocystis* sp. PCC 6803 mutant cells (e.g., R7Lα and R17Lβ) with mutations on the cytoplasmic side of Cyt *b*_559_ in PSII showed significant inhibition of the effects of blue–green light-induced NPQ and apparent acceleration on its recovery (Chiu et al., 2013). These results are consistent with the proposal that the mutations on Cyt *b*_559_ may alter the interaction between the phycobilisome and PSII reaction centers, thereby affecting the regulation of photosynthetic light harvesting in *Synechocystis* sp. PCC 6803.

## Conclusion and Perspectives

Site-directed mutagenesis studies combined with spectroscopic and functional characterization have revealed multiple roles of Cyt *b*_559_ in the assembly and photoprotection of PSII reaction centers. The findings provide convincing evidence for the physiological role(s) of Cyt *b*_559_ in a photoprotective secondary electron transfer pathway within PSII reaction centers, as was suggested from earlier studies of isolated PSII complexes (reviews in Whitmarsh and Pakrasi, 1996; Stewart and Brudvig, 1998; Shinopoulos and Brudvig, 2012). In the near future, site-directed mutagenesis studies combined with advanced high-resolution protein crystallography and spectroscopic and functional analysis will provide further new insights (e.g., structure and function relationships for different redox forms of Cyt *b*_559_) and possibly the final proof of the molecular mechanisms of Cyt *b*_559_ in PSII.

## Author Contributions

H-AC wrote the major part of the manuscript. Y-FC wrote the minor part of the manuscript, contributed the **Figure 1** and edited the references.

## Conflict of Interest Statement

The authors declare that the research was conducted in the absence of any commercial or financial relationships that could be construed as a potential conflict of interest.

## ABBREVIATIONS

APC: allophycocyanin
Car: β-carotene
Cyt *b*_559_: cytochrome *b*_559_
HP: high potential
IP: intermediate potential
LP: low potential
NPQ: non-photochemical fluorescences quenching
PQ: plastoquinone
PQH_2_: plastoquinol
PSII: Photosystem II
Q_A_: primary quinone electron acceptor in PSII
Q_B_: the secondary quinone electron acceptor in PSII
Q_C_: the third plastoquinone-binding site in PSII.
